# DNA Repair–Related Gene Signature in Predicting Prognosis of Colorectal Cancer Patients

**DOI:** 10.3389/fgene.2022.872238

**Published:** 2022-04-11

**Authors:** Min-Yi Lv, Wei Wang, Min-Er Zhong, Du Cai, Dejun Fan, Cheng-Hang Li, Wei-Bin Kou, Ze-Ping Huang, Xin Duan, Chuling Hu, Qiqi Zhu, Xiaosheng He, Feng Gao

**Affiliations:** ^1^ Department of Colorectal Surgery, The Sixth Affiliated Hospital, Sun Yat-sen University, Guangzhou, China; ^2^ Department of Gastrointestinal Endoscopy, The Sixth Affiliated Hospital, Sun Yat-sen University, Guangzhou, China; ^3^ Biomedical Big Data Center, Huzhou Maternity & Child Health Care Hospital, Huzhou, China; ^4^ Guangdong Provincial Key Laboratory of Colorectal and Pelvic Floor Diseases, Supported by National Key Clinical Discipline, Guangdong Institute of Gastroenterology, Guangzhou, China; ^5^ The University of Hong Kong, Hong Kong, Hong Kong SAR, China

**Keywords:** DNA repair–related genes, prognostic, colorectal cancer, immunotherapy, microsatellite instability

## Abstract

**Background:** Increasing evidence have depicted that DNA repair–related genes (DRGs) are associated with the prognosis of colorectal cancer (CRC) patients. Thus, the aim of this study was to evaluate the impact of DNA repair–related gene signature (DRGS) in predicting the prognosis of CRC patients.

**Method:** In this study, we retrospectively analyzed the gene expression profiles from six CRC cohorts. A total of 1,768 CRC patients with complete prognostic information were divided into the training cohort (*n* = 566) and two validation cohorts (*n* = 624 and 578, respectively). The LASSO Cox model was applied to construct a prediction model. To further validate the clinical significance of the model, we also validated the model with Genomics of Drug Sensitivity in Cancer (GDSC) and an advanced clear cell renal cell carcinoma (ccRCC) immunotherapy data set.

**Results:** We constructed a prognostic DRGS consisting of 11 different genes to stratify patients into high- and low-risk groups. Patients in the high-risk groups had significantly worse disease-free survival (DFS) than those in the low-risk groups in all cohorts [training cohort: hazard ratio (HR) = 2.40, *p* < 0.001, 95% confidence interval (CI) = 1.67–3.44; validation-1: HR = 2.20, *p* < 0.001, 95% CI = 1.38–3.49 and validation-2 cohort: HR = 2.12, *p* < 0.001, 95% CI = 1.40–3.21). By validating the model with GDSC, we could see that among the chemotherapeutic drugs such as oxaliplatin, 5-fluorouracil, and irinotecan, the IC50 of the cell line in the low-risk group was lower. By validating the model with the ccRCC immunotherapy data set, we can clearly see that the overall survival (OS) of the objective response rate (ORR) with complete response (CR) and partial response (PR) in the low-risk group was the best.

**Conclusions:** DRGS is a favorable prediction model for patients with CRC, and our model can predict the response of cell lines to chemotherapeutic agents and potentially predict the response of patients to immunotherapy.

## Background

With the third highest incidence rate in the world, colorectal cancer (CRC) is a serious threat to human health (Bray et al., 2018). Nowadays, due to lifestyle changes, there is an increasingly high incidence of mortality from CRC (Zheng et al., 2014). As one of the most common gastrointestinal tumors in general surgery, CRC is a multifactorial disease with extremely complex pathogenesis (Migliore et al., 2011). At present, the early diagnosis of CRC has involved epigenetics, genomics, and so on (Marcuello et al., 2019). DNA repair is a series of processes by which a cell recognizes and corrects damage to the DNA molecules that encode its genome (Zinovkina, 2018; Burdak-Rothkamm and Rothkamm, 2021), and it is extremely important for maintaining the stability of the genome and protecting the genome from damage by endogenous and environmental agents (Friedberg, 2001). It is estimated that human cells suffer more than $2\times 10^{4}$ DNA damage events per day (Lindahl and Wood, 1999), but generally speaking, cells can respond to this damage through efficient and highly regulated DNA repair mechanisms (Lindahl and Wood, 1999; Iyama and Wilson, 2013). Repair mechanisms include nuclear excision repair, base excision repair, mismatch repair (MMR), and double-strand break repair (Iyama and Wilson, 2013). As we all know, genomic instability caused by the destruction of DNA damage and repair mechanism can lead to cancer progression, and DNA repair genes are often found to mutate in cancer (Knijnenburg et al., 2018). Recently, Knijnenburg et al. (2018) discovered mutations related to DNA damage response genes by analyzing the TCGA data and found that several mutations in DNA damage response and repair genes occur in the colon adenocarcinoma and rectal adenocarcinoma data sets.

Due to the limited options for capturing the molecular heterogeneity of the disease and the lack of consideration and sufficient validation of other gene expressions, few of the prognostic models of early stage CRC have been applied in clinical practice (Guinney et al., 2015; Phipps et al., 2015). Thus, an accurate method is needed to identify effective prognostic models to assess the disease-free survival (DFS) of patients with CRC. The aim of the present study is to examine the interrelationships between DNA repair–related genes (DRGs) and CRC, to determine an effective prognostic model to evaluate the DFS of patients with CRC and provide guidance for clinicians in early diagnosis and treatment.

## Materials and Methods

### Patients

We retrospectively analyzed the gene expression profiles of CRC samples from six public cohorts. Totally, 1,768 samples were available for analysis in the current study. The CIT/GSE39582 (*n* = 566) was used for training the model, and The Cancer Genome Atlas colorectal cancer (TCGA, *n* = 624) was selected to serve as a validation-1 cohort. The remaining four microarray data sets (GSE14333, GSE33113, GSE37892, and GSE39084) were merged into a validation-2 cohort (*n* = 578) (Table 1). The transcriptome RNA-sequencing data of the CRC samples were from the TCGA data portal, and other microarray data sets were acquired directly from the GEO database. The institutional review board of our hospital approved this study, and data were collected from 12 May to 10 October 2020.

**TABLE 1:** Characteristics of cohorts included in this study.

Characteristics	Training cohort GSE39582	Validation-1 TCGA	Validation-2 (combination of GSE14333, GSE33113, GSE37892, and GSE39084)
Number of patients	566	624	578
Mean age	66.85	66.27	66.37
Gender			
Male	256 (45.23%)	292 (46.79%)	270 (46.71%)
Female	310 (54.77%)	332 (53.21%)	308 (53.29%)
TNM stage			
Stage I	37 (6.54%)	105 (16.83%)	53 (9.17%)
Stage II	264 (46.64%)	230 (36.86%)	280 (48.44%)
Stage III	205 (6.22%)	180 (28.85%)	164 (28.37%)
Stage IV	60 (10.60%)	88 (14.10%)	81 (14.01%)
NA	-	21 (3.37%)	-
Tumor location			
Left	342 (60.42%)	354 (56.73%)	269 (46.54%)
Right	224 (39.58%)	270 (43.27%)	216 (37.73%)
NA	-	-	93 (16.09%)
RFS event			
Yes	177 (30.62%)	100 (16.03%)	130 (22.50%)
No	380 (65.74%)	416 (66.67%)	382 (66.09%)
NA	9 (1.56%)	108 (17.31%)	66 (11.42%)
MMR status			
MSI	75 (13.25%)	189 (30.29%)	44 (7.61%)
MSS	444 (78.45%)	431 (69.07%)	114 (19.72%)
NA	47 (8.30%)	4 (0.64%)	420 (72.66%)
CIMP status			
Positive	91 (16.07%)	NA	39 (6.75%)
Negative	405 (71.56%)	NA	118 (20.42%)
NA	70 (12.37%)	624 (100%)	421 (72.84%)
TP53 status			
Wide-type	161 (28.45%)	-	39 (6.75%)
Mutation	190 (33.57%)	-	29 (5.02%)
NA	215 (37.99%)	-	510 (88.24%)
KRAS status			
Wide-type	328 (57.95%)	34 (5.45%)	110 (19.03%)
Mutation	217 (38.34%)	30 (4.81%)	48 (8.30%)
NA	21 (3.71%)	560 (89.74%)	420 (72.66%)
BRAF status			
Wide-type	461 (81.45%)	32 (5.13%)	133 (23.01%)
Mutation	51 (9.01%)	3 (0.48%)	25 (4.33%)
NA	54 (9.54%)	589 (94.39%)	420 (72.66%)

### Construction and Validation of DNA Repair–Related Gene Signature

Firstly, a complete list of DRGs was available online from MSigDB (version 6.2, https://www.gsea-msigdb.org/gsea/msigdb). We identified a list of candidate genes differentially expressed between relapsed samples and non-relapsed samples by using the “limma” R package (Diboun et al., 2006). The genes with an absolute log2-fold change of more than 1 and an adjusted *p* < 0.05 were considered for subsequent analysis. In order to minimize over-fitting risk, we applied a Cox proportional hazards regression model on CRC samples combined with the least absolute shrinkage and selection operator (LASSO) (Tibshirani, 1997). The penalty parameter was estimated by 10-fold cross-validation in the training data set at the minimum partial likelihood deviance.

We divided patients into high-risk and low-risk groups by determining the optimal threshold through the time-dependent receiver operating characteristic (ROC) curve (survivalroc, version 1.0.3) at 5 y in the training data set. The ROC curve was estimated by the Kaplan–Meier estimation method. We performed univariate and multivariate Cox regression analyses of the cohort to verify that the 11-DRG signature was independent of other clinical features.

### Functional Annotation Analysis

To evaluate the biological functions of the DNA repair–related gene signature (DRGS), enrichment analysis for differentially expressed genes in different groups was applied using the R package “gProfileR.” We used the Bioconductor package “HTSanalyzeR” to perform Gene Set Enrichment Analysis (GSEA) to predict significant dysregulated pathways (Subramanian et al., 2005; Wang et al., 2011). Gene sets of cancer hallmarks from MSigDB (Liberzon et al., 2015) were examined.

### Validation of Genomics of Drug Sensitivity in Cancer Database, Immunotherapy Database, and Tumor Immune Dysfunction and Exclusion

To further explore the clinical application of our model, we used Genomics of Drug Sensitivity in Cancer (GDSC) to analyze the differences of chemotherapeutic drugs between the high-risk group and the low-risk group.

As known, immunotherapy is a hot topic, and we want to know whether this model can predict immunotherapy. We verified our model by using the data provided in the article “Interplay of somatic alterations and immune infiltration modulates response to PD-1 blockade in advanced clear cell renal cell carcinoma (ccRCC)” published in *Nature Medicine* (Braun et al., 2020). We constructed the DRGS in the data set of advanced clear cell renal cell carcinoma and divided it into the high-risk and low-risk groups according to the cutoff of our original model. The overall survival (OS) curve was drawn using the Kaplan–Meier method. In addition, we selected some immune-related indicators in the data set and compared the differences of these indicators between the high- and low-risk groups by *t*-test. Besides, we also analyzed the OS curve of the objective response rate (ORR) of immunotherapy.

The tumor immune dysfunction and exclusion (TIDE) algorithm can be used to predict the tumor response to immune checkpoint inhibition treatment and the function of genes regulating tumor immunity, so as to effectively predict the effect of immune checkpoint inhibition treatment.

### Statistical Analysis

All the statistical analyses were performed on R (version 3.4.3, www.r-project.org). The hazard ratios were calculated using the “survcomp” package28 (version: 1.28.4) (Schröder et al., 2011). The LASSO regression was implemented using “glmnet” R package (version: 2.0.16). Cox regression analysis was used for single-factor and multifactor analyses of the results, and the receiver operating characteristic (ROC) curve and C-index were used to evaluate the model. A *p-*value of less than 0.05 was defined as statistical significance in all tests.

## Results

### Construction and Definition of the DNA Repair-Related Gene Signature

**FIGURE 1:**
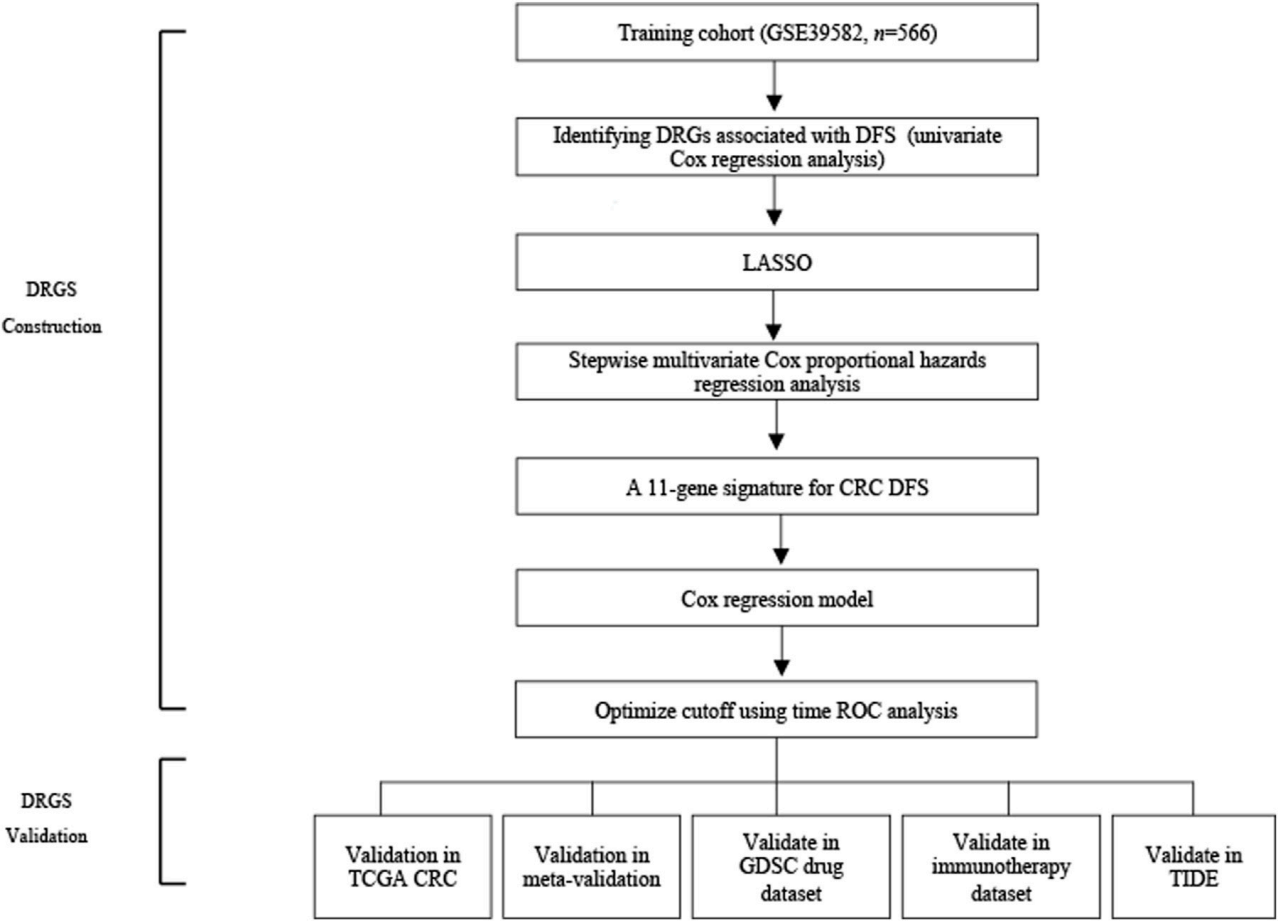
Schematic flow chart of the study.

**FIGURE 2:**
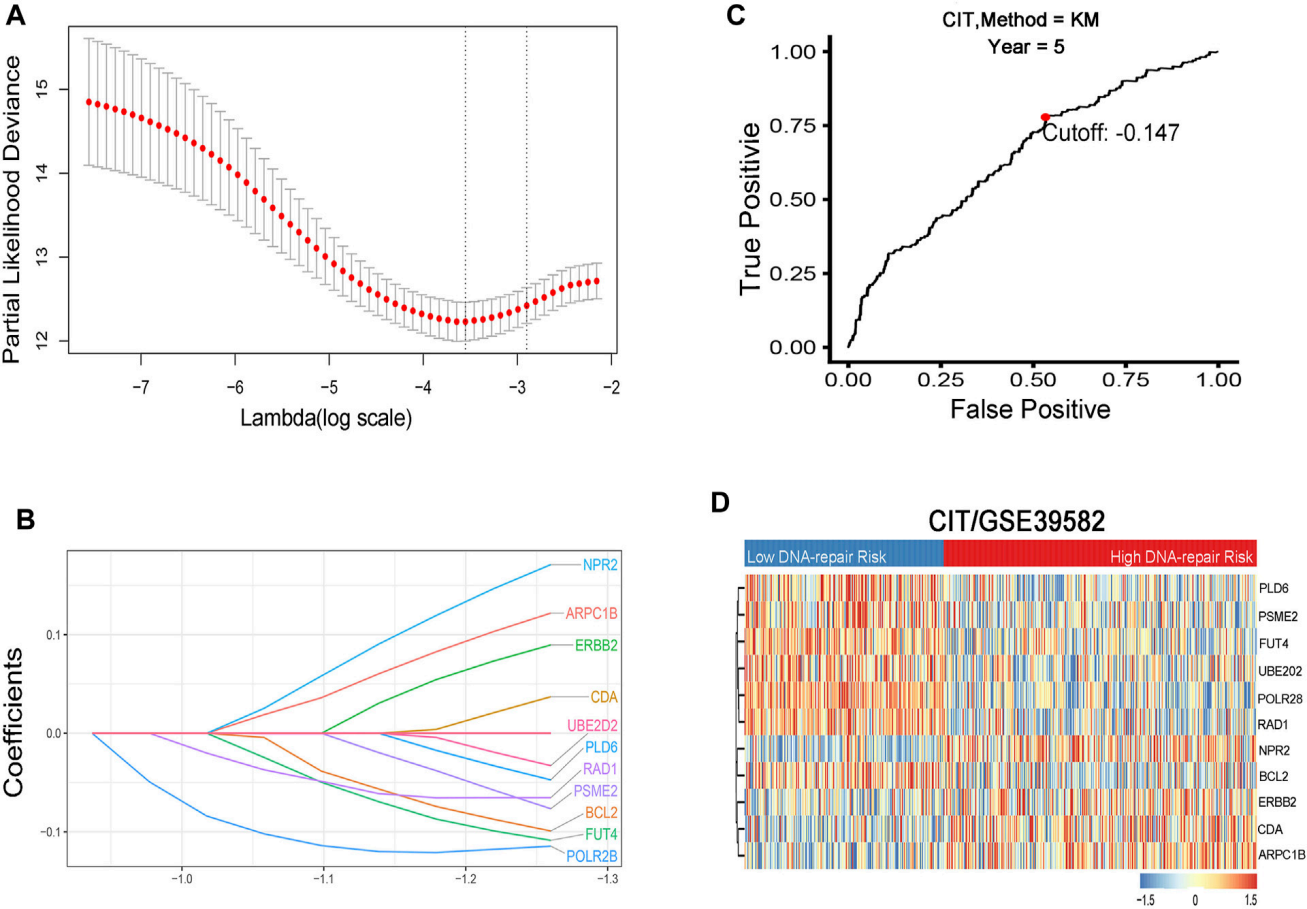
**(A)** Identification and selection of prognostic genes by LASSO Cox proportional hazards regression. **(B)** Establishment of 11 DNA repair–related genes signature from the LASSO COX regression. **(C)** Optimal cutoff point of the prognostic gene signature at 5-y OS endpoint from the ROC curve. **(D)** Heat map of the 11 DNA repair–related genes in two risk groups.

A total of 1,768 CRC patients were included in the analysis. The CIT data set (GSE39582, *n* = 566) was used as the training cohort and genes with relatively high variation were maintained as candidates (Table 1, Figure 1). With median absolute deviation >0.5 and excluding the genes expressed less in the median expression level, 1,286 genes were screened out of 1,376 DRGs measured on all platforms from the data sets. In addition, in order to improve the robustness of the identification for the limited sample size, we further selected DRGs by using the Cox proportional hazards regression against 1,000 randomized trials (80% portion of samples each time) to assess the correlation between each candidate gene and patients’ DFS in the training cohort. A total of 46 DRGs were robustly associated with individual patients’ DFS. In order to minimize the over-fitting risk, we applied a Cox proportional hazards regression model to the CRC samples combined with the LASSO. By using LASSO Cox regression, 11 prognostic DRGs were selected and combined for the construction of DRGS (Figures 2A,B). The risk scores were calculated by the formula designed by the Cox regression model. The total risk score was imputed as follows (−0.1145 × POLR2B) + (−0.0653 × RAD1) + (0.0370 × CDA) + (0.1711 × NPR2) + (−0.0328 × UBE2D2) + (−0.0992 × BCL2) + (−0.0473 × PLD6) + (0.0896 × ERBB2) + (0.1220 ×ARPC1B) + (−0.1086 × FUT4) + (−0.0765 × PSME2). The time-dependent ROC curve analysis showed that the optimal cutoff to stratify high- and low-risk groups was −0.147 (Figure 2C).

### Prognostic Evaluation of the DNA Repair-Related Gene Signature

**FIGURE 3:**
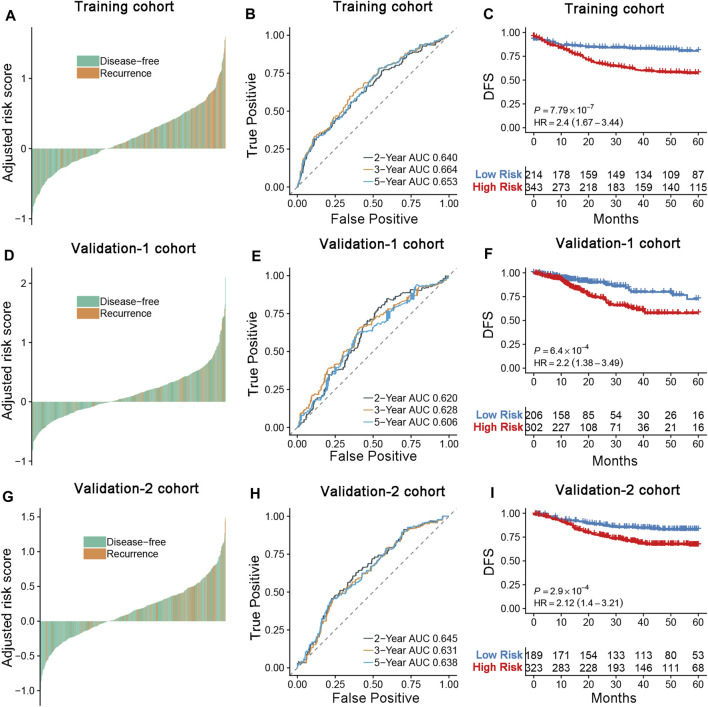
**(A,D,G)** Distribution of the DRGS risk score and its correlation to recurrence in the training, validation-1, and validation-2 cohort. **(B,E,H)** Time-dependent ROC analysis of disease-free survival for CRC patients in the training, validation-1, and validation-2 cohorts at the time points of 2, 3, and 5 y. **(C,F,I)** Kaplan–Meier curves comparing survival of patients within the low- and high-risk groups in the training cohort, validation-1, and validation-2 cohorts. *p*-values were calculated using log-rank tests.

Six colorectal cancer transcription data sets containing prognostic data were selected to assess the prognostic ability of the DRGS. The GSE39582 data set (*n* = 566) was used as a training data set (Figure 2D). The TCGA CRC dataset was enrolled as validation-1 cohort (*n* = 624), and additional data sets from the GEO were combined as validation-2 cohort (*n* = 578). Among the patients in the training and validation cohorts, more recurrences were found in the high-risk group than in the low-risk group (Figures 3A,D,G). When applied to a follow-up duration, the promising prognostic values of 2-, 3-, and 5-year AUC were 0.640, 0.664, and 0.653, respectively, in the training cohort. In the validation-1 cohort, the values of 2-, 3-, and 5-year AUC were 0.620, 0.628, and 0.606, respectively. Furthermore, in the validation-2 cohort, the values of 2-, 3-, and 5-year AUC were 0.645, 0.631, and 0.638, respectively (Figures 3B,E,H). The DRGS significantly stratified patients into the high- and low-risk groups in the training cohort (HR = 2.40, 95% CI = 1.67–3.44, *p* < 0.001), validation-1 cohort (HR = 2.20, *p* < 0.001, 95% CI = 1.38–3.49), and validation-2 cohort (HR = 2.12, *p* < 0.001, 95% CI = 1.40–3.21) (Figures 3C,F,I). Besides, the OS in the low-risk group was better than in the high-risk group (Supplementary Figure 1).

Compared to the risk scores calculated using the FDA-approved assay Oncotype DX colon algorithm, we found that the DRGS achieved better survival correlation in the training cohort (C-index, 0.78 vs 0.60), validation-1 cohort (C-index, 0.65 vs 0.51), and validation-2 cohort (C-index, 0.66 vs 0.62) (Table 2).

**TABLE 2:** C-index for DRGS risk compared with Oncotype DX.

Cohorts	DNA repair risk		Oncotype DX colon	
	C-index	95% CI	C-index	95% CI
Training cohort	0.78	0.69–0.86	0.60	0.52–0.68
Validation-1 cohort	0.65	0.51–0.79	0.51	0.37–0.65
Validation-2 cohort	0.66	0.55–0.76	0.62	0.53–0.70

To further investigate whether the DRGS could serve as an independent predictor of prognosis, univariate and multivariate Cox proportional hazards regression analyses were performed. As expected, age, sex, tumor stage, tumor location, and pathologic gene status were associated with outcomes for CRC patients (Table 3). In the univariate analysis, DRGS, MMR status, and KRAS mutation status were significantly correlated with worse prognosis in the training cohort. After adjusting for clinical features such as age, gender, tumor location, and molecular types, the DRGS remained an independent prognostic factor in the multivariate analyses in both validation cohorts.

**TABLE 3:** Univariate and multivariate analyses of DRGS, and clinical and pathologic factors.

Characteristic	Training cohort GSE39582				Validation-1 TCGA				Validation-2 (GSE14333,GSE33113,GSE37892, and GSE39084)			
	Univariate		Multivariate		Univariate		Multivariate		Univariate		Multivariate	
	HR (95%CI)	*P*	HR (95%CI)	*P*	HR (95%CI)	*P*	HR (95%CI)	*P*	HR (95%CI)	*P*	HR (95%CI)	*P*
DRGS	2.40 (1.67–3.44)	9.40E-07	1.80 (1.22–2.64)	0.0028	2.20 (1.38–3.49)	<0.001	1.85 (1.13–3.02)	0.015	2.12 (1.40–3.21)	<0.001	1.75 (1.15–2.65)	0.0085
Gender	1.21 (0.89–1.64)	0.21	-	-	1.25 (0.82–1.89)	0.3	-	-	1.06 (0.74–1.50)	0.77	-	-
Age	1.00 (0.99–1.01)	1	-	-	0.99 (0.97–1.00)	0.16	-	-	0.99 (0.98–1.00)	0.16	-	-
Tumor location	1.27 (0.92–1.74)	0.14	-	-	0.88 (0.58–1.33)	0.55	-	-	1.19 (0.81–1.75)	0.38	-	-
MMR status	2.76 (1.46–5.24)	0.0012	1.94 (0.97–3.87)	0.062	0.94 (0.61–1.45)	0.77	-	-	1.74 (0.80–3.77)	0.15	-	-
CIMP status	0.71 (0.44–1.15)	0.16	-	-	-	-	-	-	0.82 (0.39–1.72)	0.6	-	-
CIN status	1.09 (0.70–1.71)	0.71	-	-	-	-	-	-	-	-	-	-
TP53 mutation	1.41 (0.99–2.03)	0.059	-	-	-	-	-	-	1.59 (0.70–3.61)	0.26	-	-
KRAS mutation	1.40 (1.03–1.91)	0.031	1.16 (0.84–1.59)	0.37	0.76 (0.31–1.83)	0.54	-	-	1.25 (0.65–2.38)	0.5	-	-
BRAF mutation	0.96 (0.54–1.69)	0.88	NA	-	0.00 (0.00–Inf)	0.46	-	-	1.61 (0.77–3.37)	0.2	-	-

### Functional Annotation of Genomics of Drug Sensitivity in Cancer

**FIGURE 4:**
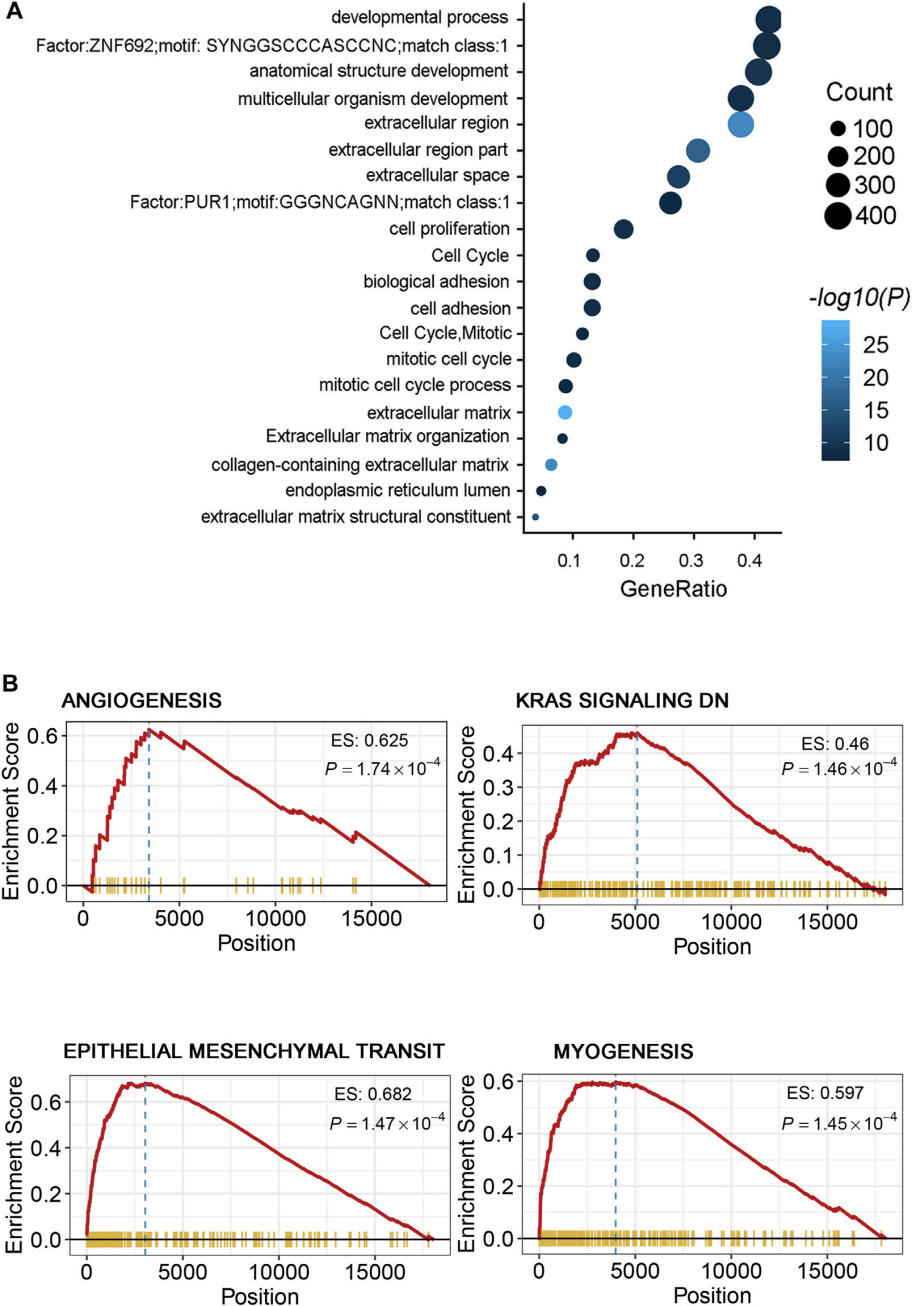
**(A)** Gene ontology of the differentially expressed genes between the two risk groups. “GeneRatio” is the percentage of total differential genes in the given GO term. **(B)** GSEA showed several metastasis-related processes enriched in the high-risk group, including angiogenesis, KRAS signaling, epithelial mesenchymal transit (EMT), and myogenesis signal pathways.

Gene Ontology (GO) analysis revealed that some biological process pathways (extracellular region, cell proliferation, and cell adhesion) were the main enriched pathways in the high-risk group (Figure 4A). In addition, the GSEA in the high-risk group when compared with the low-risk groups shown that the metastasis-related pathways (i.e., angiogenesis, KRAS signaling, epithelial mesenchymal transit, and myogenesis pathways) were enriched in the high-risk group (Figure 4B, Supplementary Table S1). Similarly, we obtained consistent results in the TCGA and validation-2 cohorts (Supplementary Figure 2). These findings suggest that the enrichment of pathways provided evidence of molecular mechanisms affected by the DRGS and thus can predict the prognosis of CRC.

### Validation of Genomics of Drug Sensitivity in Cancer Database and Immunotherapy Database

**FIGURE 5:**
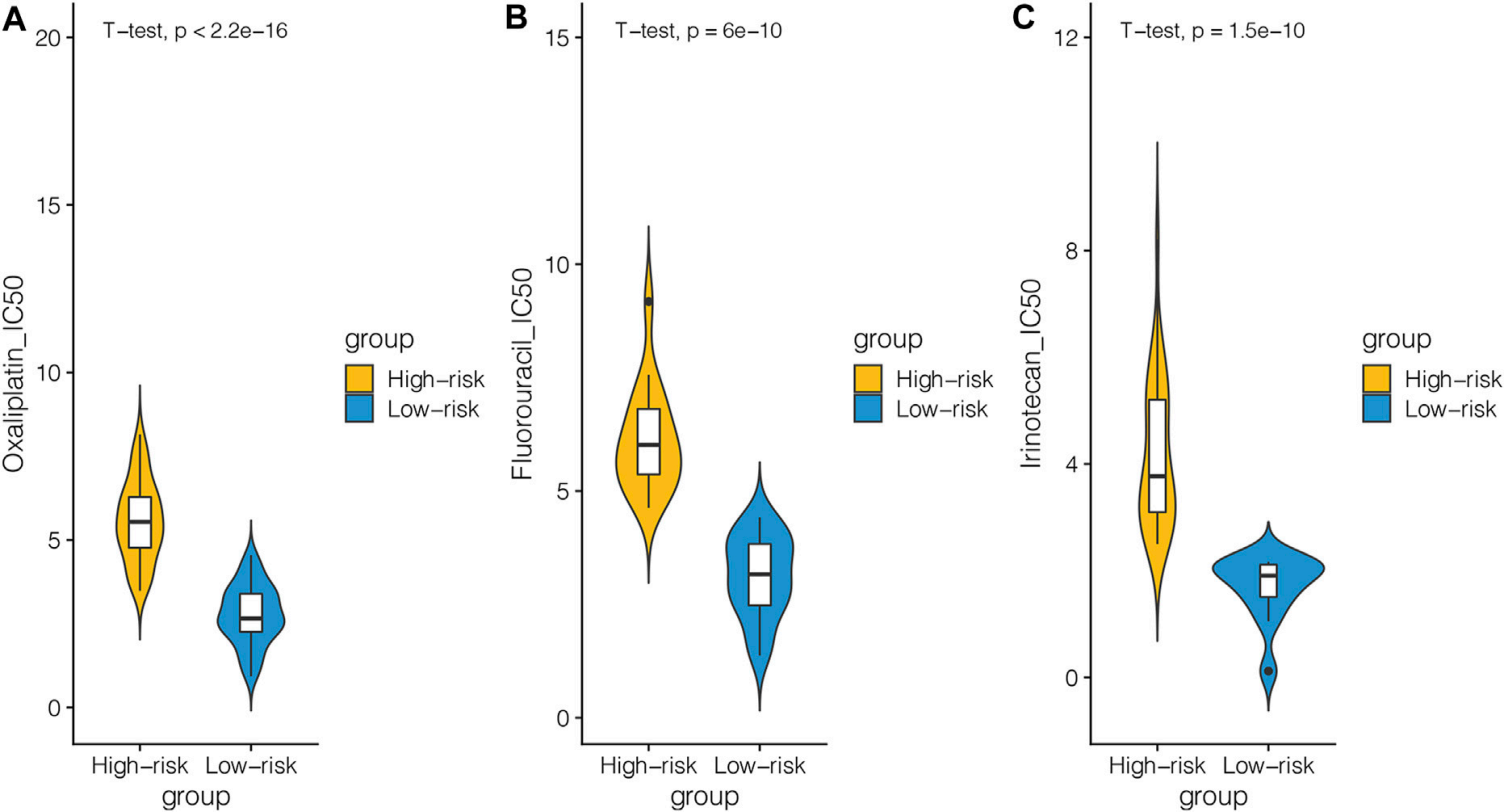
CRC cell lines in the GDSC database were divided into the high-risk and low-risk groups based on DNA repair–related signature and the differences in response to chemotherapies between the two groups were analyzed. **(A)** Relationship between the cell line of the high-risk and low-risk groups and IC50 of oxaliplatin. **(B)** Relationship between the cell line of the high-risk and low-risk groups and IC50 of fluorouracil. **(C)** Relationship between the cell line of the high-risk and low-risk groups and IC50 of irinotecan.

As known, MSI/MMR-deficient (dMMR) is widely considered as a promising biomarker, suggesting greater efficacy for immune checkpoint inhibitor (ICB) (Zhao et al., 2019). In order to further investigate the clinical application of our model, we used GDSC to analyze the differences of chemotherapeutic drugs between the high-risk and low-risk groups. We selected 48 kinds of cell lines related to CRC. After dividing the cell lines into the high-risk and low-risk groups according to the cutoff of our model, we selected the chemotherapeutic drugs commonly used in clinics to see the IC50 of the cell lines in the high-risk and low-risk groups. We can see that among the chemotherapeutic drugs such as oxaliplatin, 5-fluorouracil, and irinotecan, the IC50 of the cell line in the low-risk group was lower (Figure 5). It showed that the cell lines in the low-risk group were more sensitive to these three drugs.

**FIGURE 6:**
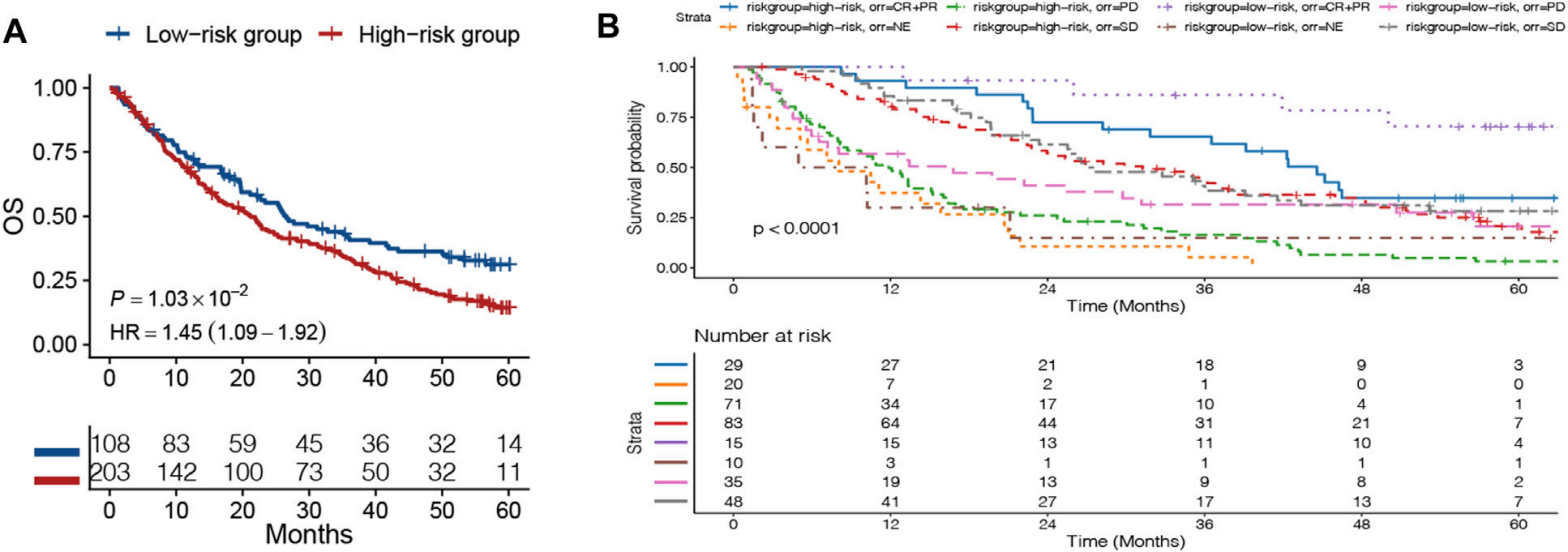
Patients in the advanced clear cell renal cell carcinoma (ccRCC) database were divided into the high-risk and low-risk groups based on the DNA repair–related signature. **(A)** Kaplan–Meier curves comparing the survival of patients within the low- and high-risk groups in the ccRCC database. **(B)** OS curve of the objective response rate (ORR) of immunotherapy in ccRCC database.

To examine whether the DRGS could predict the survival for ccRCC patients, the patients were divided into the high-risk and the low-risk groups according to the cutoff of our original model. The cutoff was still −0.147, and the prognosis data of these patients were analyzed. The OS of the high-risk group was worse than that of the low-risk group in ccRCC patients (HR = 1.45, 95% CI = 1.09–1.92, *p* = 0.0103) (Figure 6A). When it comes to the ORR of immunotherapy, we can clearly see the ORR with complete response (CR) and partial response (PR) that had better OS for both the high-risk and low-risk groups (*p* < 0.001). Notably, the OS of the low-risk group with the CR + PR was the best (Figure 6B).

### Validation of Tumor Immune Dysfunction and Exclusion Database

**FIGURE 7:**
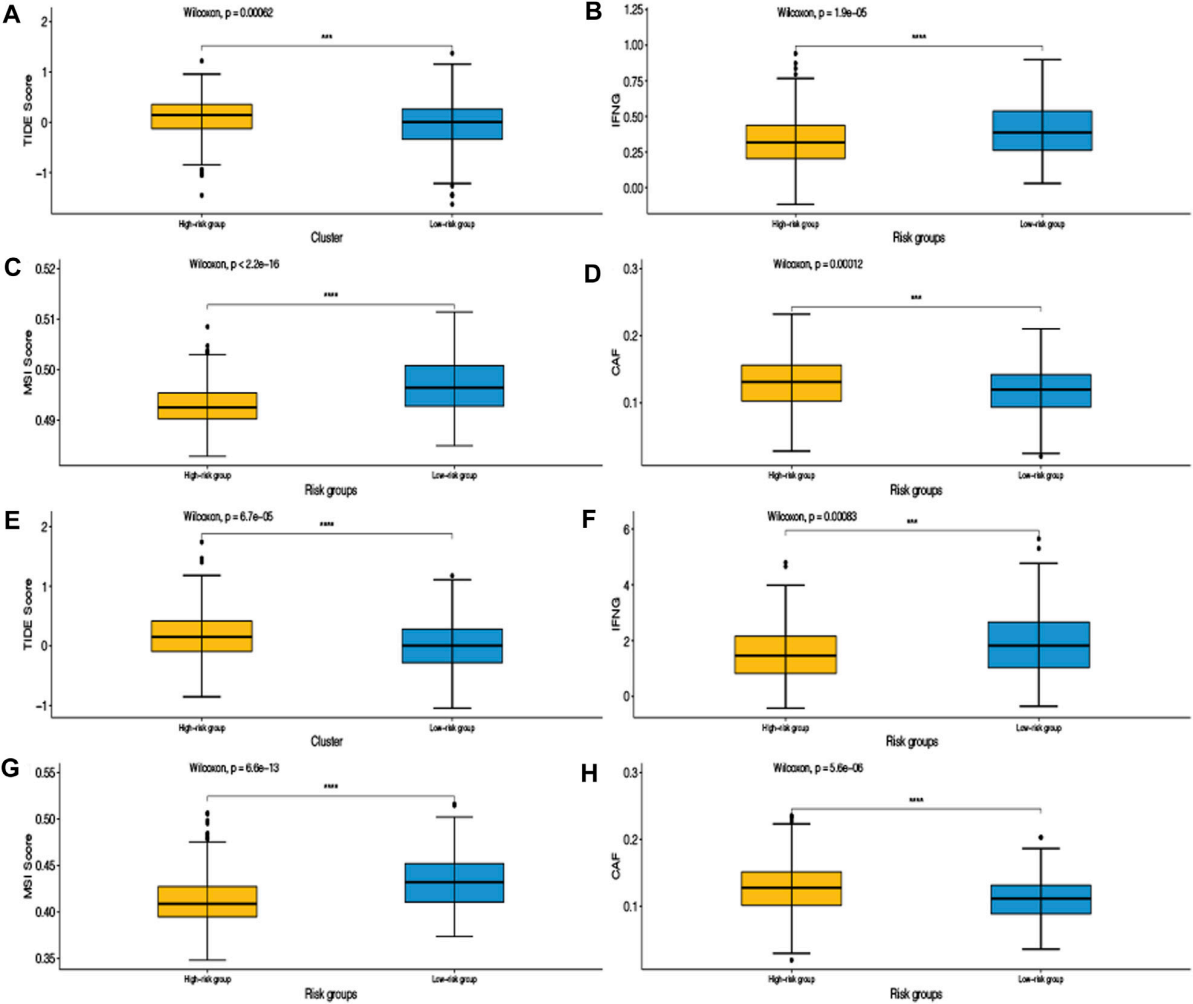
Tumor immune dysfunction and exclusion (TIDE) algorithm was validated in the training set GSE39582 **(A,B,C,D)** and the validation set TCGA **(E,F,G,H)**.

We applied the TIDE algorithm which can predict the response to immunotherapy. The low-risk group had a lower TIDE score in GSE39582 and TCGA data sets, indicating that this subgroup was most likely to benefit from immunotherapy. Besides, the low-risk group had higher interferon gamma (IFNG), higher microsatellite instability (MSI) score, and lower cancer-associated fibroblasts (CAFs) amount, which confirmed the more activated immune landscape in this subgroup (Figure 7).

## Discussion

Colorectal cancer is the leading cause of death among gastrointestinal cancers. The incidence and mortality from colorectal cancer are increasing year by year, and its prognosis is closely related to early diagnosis (Siegel et al., 2016; Siegel et al., 2017). Numerous studies have highlighted the biomarkers that are associated with the pathogenesis and biology of CRC (Shah et al., 2014; De Rosa et al., 2016; Lech et al., 2016; Das et al., 2017), and many multigene prognostic signatures have been developed for CRC (Shah et al., 2014; Kandimalla et al., 2018; Ozawa et al., 2018; Gao et al., 2019; Kandimalla et al., 2019). Unfortunately, the accuracy of their prognosis predictions remains uncertain (Fung et al., 2014). We still need much more effort to achieve good prognostic CRC prediction, which is still considered a challenge.

In recent years, some studies have found some new results in DNA pathway repair and DRGs research. DRGs inactivation may disrupt genomic integrity, which may increase the risk of accumulation of gene mutations associated with cancer development (Bouwman and Jonkers, 2012). MSI/dMMR is widely considered as a promising biomarker, suggesting greater efficacy for ICB despite some limitations (Zhao et al., 2019). In this study, our purpose was to identify and validate a reliable DRGS and improve the accuracy of survival prediction for CRC patients.

A total of 1,768 CRC patients from one training cohort and two validation cohorts were included in this study. Our prognostic DRGS can stratify CRC patients into two groups with different survival outcomes. A multivariate analysis suggested that the DRGS remained an independent prognostic factor and was significantly associated with poor prognosis in CRC. Furthermore, the C-index results of the DRGS showed its clinical superiority to Oncotype DX. Thus, it offers a significantly promising prognostic biomarker potential compared to the clinicopathological risk factors that are currently in use. The GSEA revealed that the metastasis-related pathways (i.e., angiogenesis, KRAS signaling, epithelial mesenchymal transit, and myogenesis pathways) were enriched in the high-risk group, all of which were well known to play a crucial role in the progression and proliferation of CRC in numerous studies (Cooks et al., 2013; De Simone et al., 2015; Lu et al., 2016). Further studies are required to clarify the effects of DNA repair in order to identify more targets and improve the prognosis of CRC patients.

In order to further investigate the clinical application of our model, we divided the CRC cell lines in the GDSC database into the high-risk group and low-risk group according to the DRGS and analyzed the differences in chemotherapy response between the two groups. We can see that among the chemotherapeutic drugs such as oxaliplatin, 5-fluorouracil, and irinotecan, the IC50 of the cell line in the low-risk group was lower. It showed that the cell lines in the low-risk group were more sensitive to these three drugs. On the contrary, the cell lines in the high-risk group were more insensitive to these three chemotherapeutic drugs. This indicated that our model could predict the response of cell lines to chemotherapeutic agents. This may provide some guidance for clinical medication.

We knew that MSI/dMMR was widely considered as a potential biomarker for predicting ICB (Zhao et al., 2019). We wanted to know whether our model can predict immunotherapy, so we verified our model by using the data provided in the article that “Interplay of somatic alterations and immune infiltration modulates response to PD-1 blockade in advanced clear cell renal cell carcinoma (ccRCC)” published in *Nature Medicine* (Braun et al., 2020). From the OS curve of the high- and low-risk groups, we could see that the OS of the high-risk group was worse in the ccRCC patients, and it suggested that our model can also well predict the OS of patients with ccRCC. When it comes to the ORR of immunotherapy, we can clearly see the ORR with CR + PR that had better OS in both the high-risk and low-risk groups. Notably, the OS of the low-risk group with CR + PR was the best. This indicated that our model can potentially predict the response of patients to immunotherapy. Our model can be used to further identify cancer patients who are more suitable for immunotherapy.

To further demonstrate that our model can predict the response to immunotherapy, we used the TIDE algorithm for validation in the training and validation data sets. From the results, we can see that the TIDE score of the high-risk group was higher than that of the low-risk group, indicating that the high-risk group was less sensitive to immunotherapy than the low-risk group. That is to say, the low-risk group was more effective for immunotherapy. IFNG, produced by T cells in the immune system and natural killer cells, is a potent viral inhibitor (Jorgovanovic et al., 2020). MSI, caused by defects in MMR genes, is an important molecular marker for prognosis and the development of adjuvant treatment regimens in colorectal and other solid tumors (Boland and Goel, 2010). CAFs are a group of activated fibroblasts with significant heterogeneity and plasticity in the tumor microenvironment, which have significant tumor-promoting functions (Chen and Song, 2019). The low-risk group had higher IFNG, higher MSI score, and lower cancer CAFs amount, which showed that the immune landscape of the low-risk group was more active. The consistent results of the training and validation data sets not only proved the reliability and robustness of our model but also proved that our model can predict the response to immunotherapy, which may bring some clinical benefits to CRC patients.

As for how to apply the model to the clinic, we can detect these 11 genes for patients. Because it is a small panel of genes, it can avoid the waste of large medical resources and reduce the problem of high diagnostic cost for patients as much as possible. By detecting the 11 small panel genes, we calculated the risk score of patients and grouped them. With the help of the prediction model, not only patients can make more favorable choices for themselves but also doctors can make better clinical decisions according to the patient’s risk score.

There are some limitations to our study. First, this is a retrospective study, although we validated the signature in independent data sets. In addition, the samples from primary tumor or metastatic disease may have inconsistent genetic heterogeneity, which could lead to sampling bias (NEJM Group, 2012; Mimori et al., 2018). In addition, systematic errors result from analyzing samples of disparate databases or the influence of measuring instruments, and not all batch effects can be eliminated based on their complexity. In verifying whether the model could predict immunotherapy response to CRC, we used immunotherapy data sets from ccRCC as there is currently a lack of data sets for public immunotherapy response to CRC. However, we also used the TIDE algorithm to further verify that our model can predict the immunotherapy benefit of patients. Therefore, we have sufficient evidence to prove that our model can predict the benefit of immunotherapy for patients. Although we investigated as many genes as possible, further clinical and pharmacological tests are needed to validate our results.

## Conclusion

In summary, our work provides an accurate prognostic approach for estimating the survival outcomes of CRC patients. Further prospective studies are needed to evaluate the clinical application of this signature for the prognosis of CRC.

## Data Availability

The data sets generated and analyzed during the current study are available in the TCGA cohort data downloaded from Broad GDAC Firehose (http://gdac.broadinstitute.org/). Other microarray data sets were acquired directly from the GEO database (https://www.ncbi.nlm.nih.gov/geo/query).

## Supplementary Material

Supplementary Figure 1
**(A)** Distribution of the DRGS risk score and its correlation to the survival status. **(B)** Time-dependent ROC analysis of overall survival for CRC patients at the time points of 5 and 10 y. **(C)** Kaplan–Meier curves comparing overall survival of patients within the low and high-risk groups. *P*-values were calculated using log-rank tests.

Supplementary Figure 2
**(A)** GSEA showed several metastasis-related processes enriched in the high-risk group in the TCGA validation dataset. **(B)** GSEA showed several metastasis-related processes enriched in the high-risk group in the validation-2 cohort.

## Abbreviations

DRGs, DNA repair-related genes; DRGS, DNA repair-related gene signature; CRC, colorectal cancer; DEGs, differentially expressed genes; HR, hazard ratio; GEO, Gene Expression Omnibus; GSEA, Gene Set Enrichment Analysis; LASSO, least absolute shrinkage and selection operator; OS, overall survival; ROC, receiver operating characteristic; and TCGA, The Cancer Genome Atlas.
