# Supplementary material for: DNA Repair–Related Gene Signature in Predicting Prognosis of Colorectal Cancer Patients
Source: Front Genet. 2022 Apr 11;13:872238. doi: 10.3389/fgene.2022.872238 (PMC9048823; doi:10.3389/fgene.2022.872238)
Supplement: Supplementary file 1 [file Table1.docx]

**Supplemental Table 1.** GSEA results for the comparison of high- vs. low- risk groups.

| **Pathways** | ***P*** | **ES** |
| --- | --- | --- |
| HEDGEHOG_SIGNALING | 0.00035 | 0.577 |
| ANGIOGENESIS | 0.00017 | 0.625 |
| MYC_TARGETS_V2 | 0.00025 | -0.600 |
| PROTEIN_SECRETION | 0.00027 | -0.544 |
| INTERFERON_ALPHA_RESPONSE | 0.00028 | -0.492 |
| ANDROGEN_RESPONSE | 0.00027 | -0.434 |
| UNFOLDED_PROTEIN_RESPONSE | 0.00028 | -0.494 |
| SPERMATOGENESIS | 0.00058 | -0.354 |
| COAGULATION | 0.00015 | 0.573 |
| DNA_REPAIR | 0.00030 | -0.405 |
| OXIDATIVE_PHOSPHORYLATION | 0.00031 | -0.513 |
| KRAS_SIGNALING_DN | 0.00015 | 0.460 |
| G2M_CHECKPOINT | 0.00030 | -0.666 |
| APICAL_JUNCTION | 0.00015 | 0.505 |
| KRAS_SIGNALING_UP | 0.00074 | 0.340 |
| E2F_TARGETS | 0.00031 | -0.721 |
| MTORC_SIGNALING | 0.00032 | -0.553 |
| MYC_TARGETS_V1 | 0.00031 | -0.671 |
| EPITHELIAL_MESENCHYMAL_TRANSIT | 0.00015 | 0.682 |
| MYOGENESIS | 0.00015 | 0.587 |
| GLYCOLYSIS | 0.00032 | -0.346 |
| ALLOGRAFT_REJECTION | 0.00156 | -0.298 |
| MITOTIC_SPINDLE | 0.00031 | -0.466 |
| INTERFERON_GAMMA_RESPONSE | 0.00031 | -0.429 |
| FATTY_ACID_METABOLISM | 0.00148 | -0.320 |
| PI3K_AKT_MTOR_SIGNALING | 0.00379 | -0.339 |
| TNFA_SIGNALING_VIA_NFKB | 0.02650 | -0.257 |
| IL6_JAK_STAT3_SIGNALING | 0.01650 | -0.327 |
| CHOLESTEROL_HOMEOSTASIS | 0.02130 | -.0338 |
