# Supplementary figures and images for: DNA Repair–Related Gene Signature in Predicting Prognosis of Colorectal Cancer Patients
Source: Front Genet. 2022 Apr 11;13:872238. doi: 10.3389/fgene.2022.872238 (PMC9048823; doi:10.3389/fgene.2022.872238)

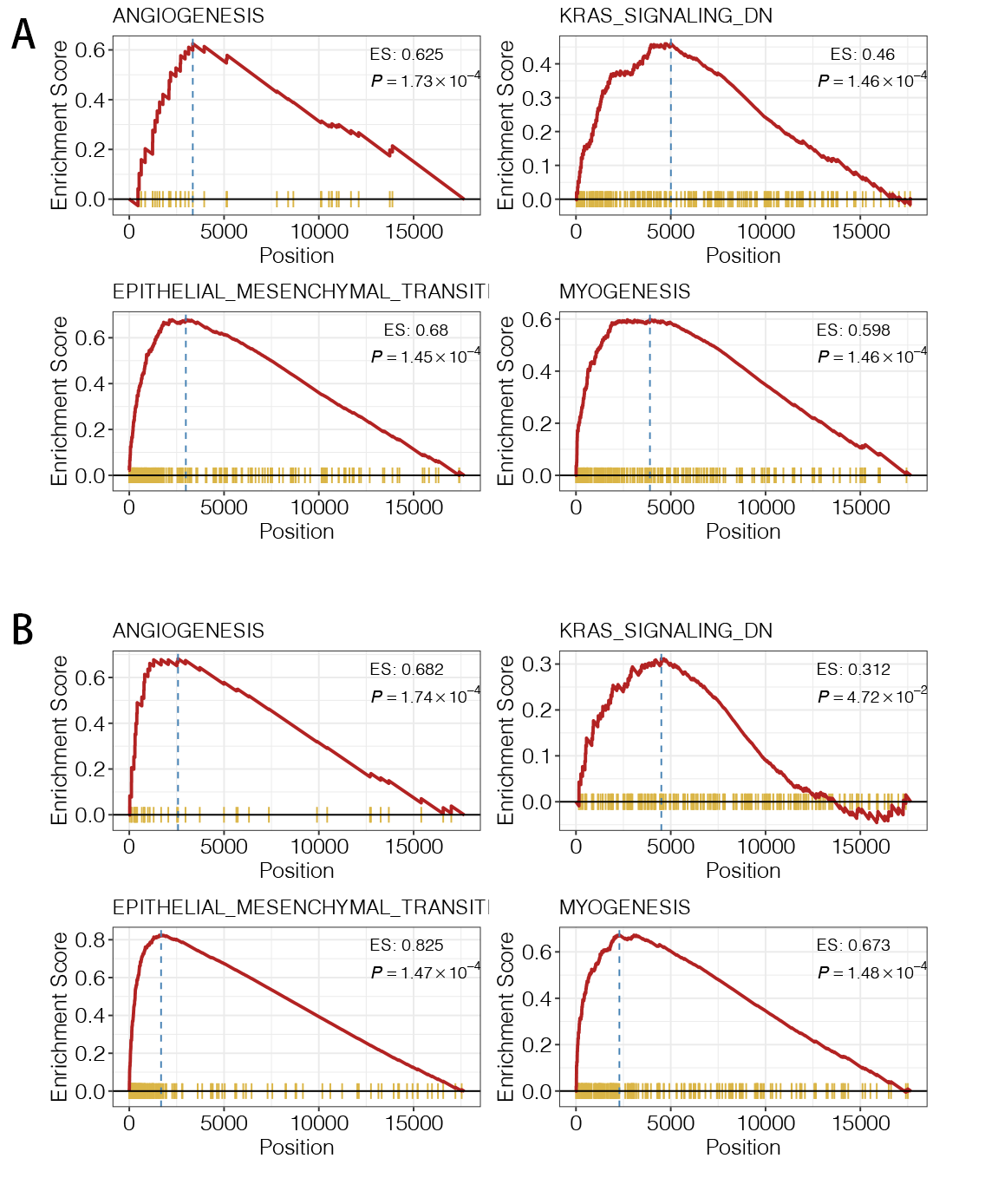

Supplement: Supplementary file 2 [file Image2.TIF]

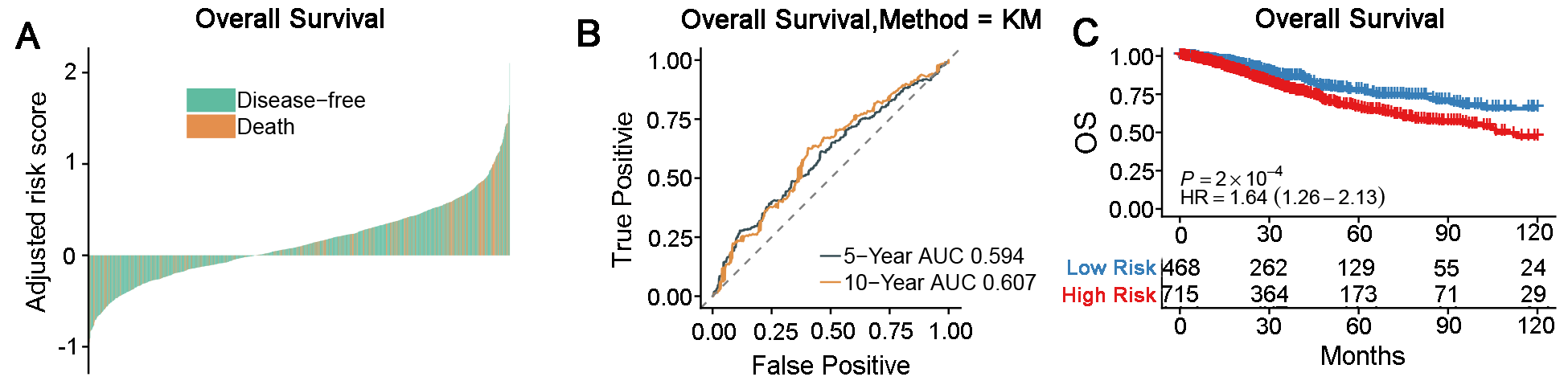

Supplement: Supplementary file 3 [file Image1.TIF]
